# Feeding whole-plant ensiled corn stover affects growth performance, blood parameters, and Cecal microbiota of Holdobagy goose

**DOI:** 10.3389/fvets.2023.1210706

**Published:** 2023-06-15

**Authors:** Xianze Wang, Guangquan Li, Huiying Wang, Yi Liu, Yunzhou Yang, Cui Wang, Shaoming Gong, Daqian He

**Affiliations:** Institute of Animal Husbandry and Veterinary Science, Shanghai Academy of Agricultural Science, Shanghai, China

**Keywords:** whole-plant ensiled corn stalks, Holdobagy geese, growth performance, alpha diversity, principal coordinate analysis, Spearman’s analysis

## Abstract

**Introduction:**

The aim of this study was to investigate the effects of adding whole-plant ensiled corn stalks (*WECS*) to the diet of Holdorbagy geese on their growth performance, serum parameters, and cecal microbiota. Geese farming is an important agricultural practice, and optimizing their diet can contribute to better growth and health outcomes. However, there is limited research on the utilization of *WECS* as a feed source for geese. Understanding the potential effects of *WECS* on growth, blood parameters, and cecal microbiota can provide valuable insights into its feasibility and impact on geese farming practices.

**Methods:**

A total of 144 six-week-old Holdorbagy geese were randomly assigned to one of three groups: a control group (0% *WECS*), a group fed 15% *WECS* and 85% concentrated feed (15% *WECS*), and a group fed 30% *WECS* and 70% concentrated feed (30% *WECS*). The trial period lasted for three weeks, during which the growth performance, serum parameters, and cecal microbiota were assessed.

**Results:**

The results revealed significant findings in different aspects. Firstly, the feed-to-gain ratio (F/G ratio) of the 15% *WECS* group was significantly higher than that of the control group (*p*<0.05), indicating potential challenges in feed efficiency. Additionally, the average daily feed intake (ADFI) of both the 15% and 30% *WECS* groups was significantly higher than that of the control group (*p*<0.05), suggesting increased appetite or palatability of the diet containing *WECS*. In terms of serum parameters, the level of lactate dehydrogenase (LDH) in the 30% *WECS* group was significantly lower than that in the control group (*p*<0.05). Moreover, there was a tendency for increasing Fe levels and decreasing Zn levels with higher levels of *WECS* supplementation, although the differences were not statistically significant (*p*<0.05). Furthermore, the principal coordinate analysis showed significant differences in the composition of cecal microbiota among the three groups (*p* < 0.01). The observed_species, Shannon, and Pielou_e indices of the 30% *WECS* group were significantly higher than those of the 0% and 15% *WECS* groups (*p*<0.05), while the Simpson index of the 15% *WECS* group was significantly lower than that of the control group (*p*<0.05).

**Discussion:**

The results indicate that the addition of *WECS* to the geese diet has both positive and negative effects. The study suggests that *WECS* can be a long-term stable feed source for geese, which can contribute to reducing feeding costs. However, it is important to monitor the amount of *WECS* added as it can affect the absorption of Zn by geese. Supplementation of Zn in the diet might be necessary to meet the needs of geese. Notably, adding 30% *WECS* to the diet can increase the richness, evenness, and diversity of the cecal microbiota, indicating potential benefits to gut health. In conclusion, this study highlights the potential of *WECS* as a feed source for geese. It provides valuable insights into the effects of *WECS* on growth performance, serum parameters, and cecal microbiota. These findings contribute to optimizing geese farming practices, improving feed utilization, and enhancing overall productivity and well-being of geese. Further research is needed to determine the optimal inclusion level of *WECS* and to explore strategies for mitigating any negative effects.

## Introduction

Geese are herbivorous waterfowl, especially in wild flocks. However, in modern intensive production systems, high-energy and high-protein concentrate feed has become the main component of meat goose diets. Although concentrate feed has high nutritional value, it is expensive. Solely feeding concentrate may bring uncontrollable nutritional diseases to goslings, thereby limiting meat goose production (1).

Therefore, people are increasingly interested in exploring unconventional feeds that can replace meat goose concentrate, reduce reliance on traditional feeds, and enhance sustainability (2). Whole-plant ensiled corn stalks (*WECS*) are one such alternative feed source. Compared to ordinary corn and ensiled straw, *WECS* have more complete and balanced nutritional components and potential benefits for animal health and welfare.

The digestive structure of geese has innate advantages in digesting high-fiber foods, such as *WECS*. Geese have a relatively independent and developed caecum, similar to the rumen of ruminants, which allows insufficiently digested food to stay inside for sufficient fermentation (3, 4). The caecum of geese contains cellulose-digesting microorganisms lacking in other avian digestive tracts, which convert cellulose into short-chain fatty acids (SCFAs) and provide energy to geese (5). In addition, the digestive tract of geese has strong motility and rotational ability, and the gizzard accounts for about 8% of their body weight (6). This helps to fully grind plant cell walls, promote the decomposition and utilization of cellulose (7).

Therefore, adding high-yield and low-cost *WECS* as an additional dietary supplement may reduce production costs and improve the intestinal health of geese. Although recent studies on using ensiled corn in meat goose production (8–10) have yielded encouraging results, the use of traditional ensiled corn stalks is mainly suitable for ruminants such as cows, beef cattle, etc., and their application in monogastric animals such as meat geese needs further research and confirmation.

Therefore, we conducted a study to explore the effects of adding different levels of *WECS* on meat goose growth performance and caecal microorganisms. The results of this study will help us understand the potential benefits and limitations of using *WECS* as meat goose feed and provide reference for their application in meat goose production.

## Materials and methods

The experimental procedures were conducted in compliance with the guidelines established by the Chinese Animal Care and Protocol Committee and approved by the Animal Care and Use Committee of the Shanghai Academy of Agricultural Sciences.

### Grouping and treatment of geese

A total of 144 six-week-old Holdorbagy geese were purchased from (Xiangtiange Poultry, Maanshan, Anhui, China). The geese were randomly divided into three treatment groups based on body weight, with each group having 8 replicates and 6 geese in each replicate. Specifically, the 0 *WECS* group was the control group, which was fed 100% commercial feed, while the 15 *WECS* group was fed a diet consisting of 15% whole-plant ensiled corn stover and 85% commercial feed, and the 30 *WECS* group was fed a diet consisting of 30% whole-plant ensiled corn stover and 70% commercial feed. The geese were raised in a net fence with dimensions of 2 meters in length, 2 meters in width, and 0.6 meters in height, and each fence was equipped with a drinking waterer and a feeding trough. The experiment lasted for 3 weeks.

### Nutritional composition of diets and silage corn

The*WECS* and concentrate feed required for the experiment were purchased from (Shengwang Feed Co., Ltd., Fengxian District, Shanghai, China). After air-drying, the dry matter, crude protein (CP), crude fiber (*CF*), ether extract (EE) and metabolizable energy (ME) of the *WECS* were measured according to the AOAC (1998) test method. Based on the *WECS* addition level, the daily feed of each group of test geese was designed according to the standards of the National Research Council (11). These feeds met the minimum nutritional requirements of geese during the growth period. The feed formula is shown in Table 1, and the nutritional level of the *WECS* is shown in Table 2.

**Table 1:** Ingredients and nutrient compositions of experimental diets (air dry basis).

Items	Diet		
	0 *WECS*	15 *WECS*	30 *WECS*
Ingredient, %			
Corn	66.07	58.25	57.18
Soybean meal (43% CP)	26.42	26.99	27.22
Soybean oil	0.00	2.45	4.00
Premix^2^	3.00	3.00	3.00
Corn gluren	4.51	4.51	5.00
Silage corn (air dry basis)	0.00	4.8	9.6
Total	100	100	100
Calculated nutrient concentration^1^			
ME MJ/Kg	11.77	11.71	11.48
CP, %	18.00	18.00	18.00
EE, %	3.00	2.89	2.82
*CF*, %	2.82	4.94	6.22
Lysine, %	1.01	1.01	1.01
Methionine, %	0.45	0.45	0.45
Cystine, %	0.40	0.40	0.40
Calcium, %	1.52	1.52	1.52
Non-phytate, %	0.40	0.40	0.40
Arginine, %	1.20	1.20	1.20

^1^Calculated nutrient concentration: Dietary nutrient composition are calculated values.

^2^Provided per kg of diet:Vitamin A 233000 IU, Vitamin B1 26.7 mg, Vitamin B2 (riboflavin) 150 mg, Pantothenic acid 5 mg, Vitamin B6 60 mg, Vitamin B12 0.3 mg, Vitamin D3 50,000 IU, Vitamin E 433.14 IU, Vitamin K3 16.7 mg, biotin 2.5 mg, folic acid 20 mg, nicotinamide 800 mg, choline chloride 10 g; Cu 0.3 mg, Fe 2.0 g, Mn 2.0 g, Zn 2.0 g, I 16.7 mg, Se 3.3 mg, Ca 4%, P 3.3%, salt 10 g. *0SC* control group, *15SC* 15% silage corn +85% concentrate feed, *30SC* 30% silage corn +70% concentrate feed.

**Table 2:** Nutrient composition of *WECS* (air dry basis).

Items	Nutrient concentration^1^
Dry matter, %	32
CP, %	8.61
ME, MJ/kg	15.91
Lysine, %	0.90
Methionine+ Cystine, %	0.66
Threonine, %	0.63
*CF*, %	22.56
EE, %	0.5

^1^Nutrient concentration: Silage corn nutrient composition is measured.

### Sample collection

In the third week of the experiment, all geese were weighed after an 8-h fast. One male goose with a weight close to the average weight was selected for each replicate group, and its brachial vein blood was collected. The collected blood was left to stand at room temperature for 2 h, then centrifuged at 3000 rpm for 10 min to separate the serum. The serum was collected and used for subsequent indicator measurements. After blood collection, the geese were intravenously injected with 5 mL of 3% pentobarbital solution, then slaughtered by bleeding from the jugular vein. The abdominal cavity of the goose was opened, and the cecum was cut open to collect its contents in a 2 mL EP tube. Finally, the collected cecal contents were rapidly frozen in liquid nitrogen and stored at −80°C for subsequent indicator measurements.

### Growth performance determination

On the day before the experiment began and ended, geese were fasted for 8 h overnight, and then weighed the next morning. The Initial Body Weight (IBW) and Final Body Weight (FBW) of each goose were recorded. The feed consumption of each replicate group was recorded during the entire experimental period, and the Average Daily Feed Intake (ADFI), Average Daily Gain (ADG), and Feed Gain ratio (F/G) were calculated.

The calculation methods of growth performance indicators were as follows:

$$\mathrm{ADG}=\left(\mathrm{FBW}–\mathrm{IBW}\right)/\mathrm{number of days in the experiment}.$$

F/G=feed consumption/total weight gain.

ADFI=F/G×ADG.

### Determination of blood parameters

The serum, which was stored at −80°C after centrifugation, was sent to (Ingle Testing Technology Service Co., Ltd., Shanghai, China) for the detection of serum calcium (Ca), phosphorus (P), zinc (Zn), and iron (Fe). At the same time, the serum stored at −80°C was also sent to (Shanghai Pinyi Biotechnology Co., Ltd., Shanghai, China) for the detection of serum ghrelin (GHRL), insulin-like growth factor-1 (IGF-1), lactate dehydrogenase (LDH), and serum amylase (AMY).

### 16S rRNA sequencing

The contents of the cecum were sent to Passino Biotechnology Co., Ltd. in Shanghai, China for 16S rRNA analysis and data processing. To put it simply, first, fecal genomic DNA extraction kits (Tiagen Biotech, Beijing, China) were used to extract DNA from the cecal contents. After assessing the quality and quantity of the extracted DNA, universal primers (F:ACTCCTACGGGAGGCAGCA and R:GGACTACHVGGTWTCTAATPCR) were used to amplify the V3-V4 region of the 16S rDNA, and a library was constructed. These libraries were then subjected to paired-end sequencing on the Illumina platform (Illumina, San Diego, California, United States) (12). The QIIME DADA2 DENOISE-PAIRED tools were used for quality control, denoising, stitching, and chimera filtering. After denoising all libraries, the ASVs (AMPLICON Sequence Variants) and ASV tables were combined, and singleton ASVs were removed.

### Statistics and analysis

All data were initially processed using Microsoft Excel 2003 (Redmond, Washington, United States) and subsequently analyzed using SPSS 17.5 (International Business Machines Corporation, Armonk, New York, United States) software, which performed one-way ANOVA and Duncan multiple comparison tests to determine the differences between the experimental groups. All data were presented as mean ± standard error of the mean (SEM). Differences with *p* < 0.05 were considered significant, while differences with *p* < 0.01 were considered highly significant. To annotate species from the phylum to genus level, we employed the classify-sklearn algorithm (13) in QIIME2 (14) software, utilizing a pre-trained Naive Bayes classifier. Furthermore, we calculated the alpha diversity index using the ggplot2 package in the R language of QIIME2 software and generated dilution and abundance grade curves using an R script. To visualize the flattened ASV table, we utilized the ape package in the R language of QIIME2 software to calculate the Bray-Curtis distance (15) and created a two-dimensional scatter plot using an R script.

## Result

### Growth performance

Table 3 indicates that there were no significant distinctions (*p* > 0.05) in the ADG, IBW, and FBW of the experimental geese in the different treatment groups. However, the F/G of the geese in the 15 *WECS* group was significantly higher than that of the 0 *WECS* group (*p* < 0.05). Moreover, the ADFI of the geese in the 15 *WECS* and 30 *WECS* groups were significantly higher than that of the 0 *WECS* group (*p* < 0.01).

**Table 3:** Effects of feeding WECS on the growth performance of geese.

Item	Diet treatment			value of *p*
	0 *WECS*	15 *WECS*	30 *WECS*	
IBW, g	2513.71 ± 29.37	2530.97 ± 29.41	2532.45 ± 30.07	0.884
FBW, g	4239.61 ± 69.39	4367.77 ± 84.61	4344.61 ± 44.31	0.458
ADG, g	82.19 ± 2.49	87.46 ± 2.97	86.29 ± 3.05	0.392
F/G	5.24 ± 0.25^a^	6.39 ± 0.37^b^	6.22 ± 0.11^ab^	0.027
ADFI, g	437.44 ± 13.19^A^	556.83 ± 18.50^B^	536.84 ± 19.28^B^	*p*<0.001

0 *WECS* control group, 15 *WECS* 15% whole-plant ensiled corn stalks +85% concentrate feed, 30 *WECS* 30% whole-plant ensiled corn stalks +70% concentrate feed.

^a–b^ Means with different superscript letters in the same row differ significantly (*p* < 0.05).

^A–B^ Means with different superscript letters in the same row differ extremely significantly (*p* < 0.01).

### Serum minerals

Based on Table 4, there were no statistically significant differences in the levels of Ca, P, Fe and Zn in the serum of geese across the different treatment groups (*p*>0.05). However, there was a tendency toward an increase in Fe levels and a decrease in Zn levels in the serum of geese in the experimental group as the proportion of *WECS* increased, although these trends were not statistically significant (*p* > 0.05).

**Table 4:** Effects of feeding *WECS* on serum mineral levels of geese.

Items	Diet treatment			value of *p*
	0 *WECS*	15 *WECS*	30 *WECS*	
Ca, mmol/L	1.64 ± 0.25	1.86 ± 0.17	1.81 ± 0.17	0.720
P, mmol/L	1.57 ± 0.21	1.85 ± 0.15	1.72 ± 0.17	0.545
Fe, μmol/L	53.41 ± 7.73	69.19 ± 5.00	76.28 ± 8.52	0.096
Zn, μmol/L	122.33 ± 24.75	103.55 ± 13.52	77.91 ± 16.68	0.275

0 *WECS* control group, 15 *WECS* 15% whole-plant ensiled corn stalks +85% concentrate feed, 30 *WECS* 30% whole-plant ensiled corn stalks +70% concentrate feed.

### Serum hormones and enzymes

As shown in Table 5, there were no significant differences in the levels of GHRL, LGF-1, and AMY in the serum of the experimental geese across the treatment groups (*p* > 0.05). Nevertheless, the feeding of *WECS* led to a reduction in the serum LDH level of the experimental geese, with a significant difference observed at 30% *WECS* addition (*p* < 0.05).

**Table 5:** Effects of feeding *WECS* on serum hormones and enzymes levels of geese.

Items	Diet treatment			value of *p*
	0 *WECS*	15 *WECS*	30 *WECS*	
GHRL, ng/mL	90.51 ± 11.73	104.57 ± 11.75	100.36 ± 26.90	0.855
IGF-1, ng/mL	99.99 ± 8.59	96.52 ± 8.12	96.26 ± 23.07	0.981
LDH, ng/mL	65.68 ± 7.02^a^	40.27 ± 7.47^ab^	29.67 ± 12.54^b^	0.044
AMY, μg/mL	13.40 ± 0.46	11.78 ± 0.55	10.94 ± 2.39	0.639

0 *WECS* control group, 15 *WECS* 15% whole-plant ensiled corn stalks +85% concentrate feed, 30 *WECS* 30% whole-plant ensiled corn stalks +70% concentrate feed.

^a–b^ Means with different superscript letters in the same row differ extremely significantly (*p* < 0.05).

### Sequence quality assessment

**Figure 1:**
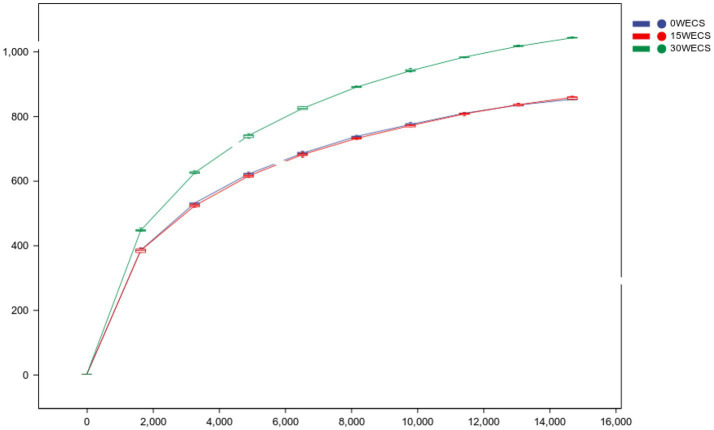
Rarefaction Curve. The abscissa is the leveling depth, and the ordinate is the median value and boxplot of the alpha diversity index calculated 10 times. 0 *WECS* control group, 15 *WECS* 15% whole-plant ensiled corn stalks +85% concentrate feed, 30 *WECS* 30% whole-plant ensiled corn stalks +70% concentrate feed.

Table 6 illustrates that the original data contained 911,642 ASVs with matching forward and reverse primers. After quality control, denoising, splicing, chimera removal, and removal of singletons, a total of 460,877 high-quality ASVs were retained, with an effective ratio of 50.55%. Figure 1 depicts the length and percentage distribution of high-quality sequences in the samples. The length of high-quality sequences ranges from 405–432 bp, with 39.77% of sequences having a length of 425 bp, and 0.25% of sequences having a length of 409 bp, which is the lowest percentage. Notably, no abnormal sequence lengths were detected.

**Table 6:** Sample sequencing volume statistics.

SampleID	Input	Filtered	Denoised	Merged	Non-chimeric	Non-singleton
0 *WECS* 1	33,538	28,677	26,942	19,945	16,895	16,710
0 *WECS* 2	33,578	29,169	28,031	23,123	18,501	18,385
0 *WECS* 3	38,856	34,113	32,744	24,367	19,668	19,418
0 *WECS* 4	39,960	34,238	32,291	24,952	22,130	21,963
0 *WECS* 5	38,104	33,337	31,865	25,528	20,717	20,471
0 *WECS* 6	34,625	29,069	27,300	19,077	17,113	16,912
0 *WECS* 7	44,965	37,540	35,156	26,221	21,088	20,712
0 *WECS* 8	34,808	29,545	28,184	21,439	17,210	16,999
15 *WECS* 1	36,690	32,005	30,337	23,243	20,446	20,272
15 *WECS* 2	37,922	32,662	31,051	24,079	19,554	19,241
15 *WECS* 3	37,072	32,544	31,421	24,967	21,371	21,205
15 *WECS* 4	37,691	32,757	31,426	24,865	20,846	20,625
15 *WECS* 5	36,976	32,361	31,052	26,055	22,361	22,247
15 *WECS* 6	37,266	32,666	31,047	23,700	19,985	19,723
15 *WECS* 7	39,791	33,769	31,609	23,130	20,534	20,319
15 *WECS* 8	45,359	39,179	37,526	29,990	25,670	25,388
30 *WECS* 1	36,613	30,755	28,998	22,792	17,491	17,189
30 *WECS* 2	40,590	34,004	31,898	22,485	18,422	18,080
30 *WECS* 3	37,109	32,481	30,706	23,207	18,451	18,133
30 *WECS* 4	36,721	30,747	29,171	23,503	18,224	17,878
30 *WECS* 5	42,928	37,316	34,803	23,881	18,732	18,267
30 *WECS* 6	38,232	33,188	30,989	22,441	17,722	17,231
30 *WECS* 7	34,688	29,468	27,609	21,337	15,676	15,464
30 *WECS* 8	37,560	32,671	30,684	22,955	18,500	18,045

The second column indicates the number of sequences that matched the forward and reverse primers in the original data. The third column displays the amount of data after removal of low-quality sequences. The fourth column shows the sequence data after denoising. The fifth column represents the sequence amount after splicing. The sixth column displays the sequence amount after removing chimeras. The seventh column represents the sequence amount after removing singleton.

### Alpha diversity

Table 7 shows the alpha diversity indices between groups, and the Goods_coverage for all test groups is >98%, indicating that the sequencing results can accurately represent the real situation of the samples. The observed_species, Shannon, and Pielou_e indices in group 30 *WECS* were significantly higher than those in groups 0 *WECS* and 15 *WECS* (*p* < 0.05), while the Simpson index in group 15 *WECS* was significantly lower than that in groups 0 *WECS* and 30 *WECS* (*p* < 0.05).

**Table 7:** Effects of *WECS* on α-diversity of cecal contents bacterial communities (*n* = 8).

Items	Diet treatment			value of *p*
	0 *WECS*	15 *WECS*	30 *WECS*	
Observed_species	853.34 ± 53.63^b^	857.35 ± 57.12^b^	1042.70 ± 56.37^a^	0.040
Shannon	7.15 ± 0.23^b^	7.09 ± 0.21^b^	7.89 ± 0.15^a^	0.016
Simpson	0.96 ± 0.01^b^	0.95 ± 0.01^a^	0.98 ± 0.00^b^	0.042
Chao1	944.52 ± 61.39	989.27 ± 76.01	1138.89 ± 63.53	0.126
Pielou_e	0.74 ± 0.02^b^	0.73 ± 0.01^b^	0.79 ± 0.01^a^	0.019
Faith_pd	42.57 ± 1.23	41.99 ± 1.80	46.75 ± 1.51	0.079
Goods_coverage	>98%	>98%	>98%	/

0 *WECS* control group, 15 *WECS* 15% whole-plant ensiled corn stalks +85% concentrate feed, 30 *WECS* 30% whole-plant ensiled corn stalks +70% concentrate feed.

^a–b^ Means with different superscript letters in the same row differ significantly (*p* < 0.05).

### Rarefaction curve

Figure 1 shows that, at the same sequencing depth, the dilution curves of the 0 *WECS* and 15 *WECS* groups are relatively flat. This suggests that the diversity and uniformity of microbial species in these two groups are similar. On the other hand, the dilution curve of the 30 *WECS* group is relatively steep and higher, indicating a greater diversity of community composition in the 30 *WECS* group.

### Composition of cecal contents at the level of phyla

**Figure 2:**
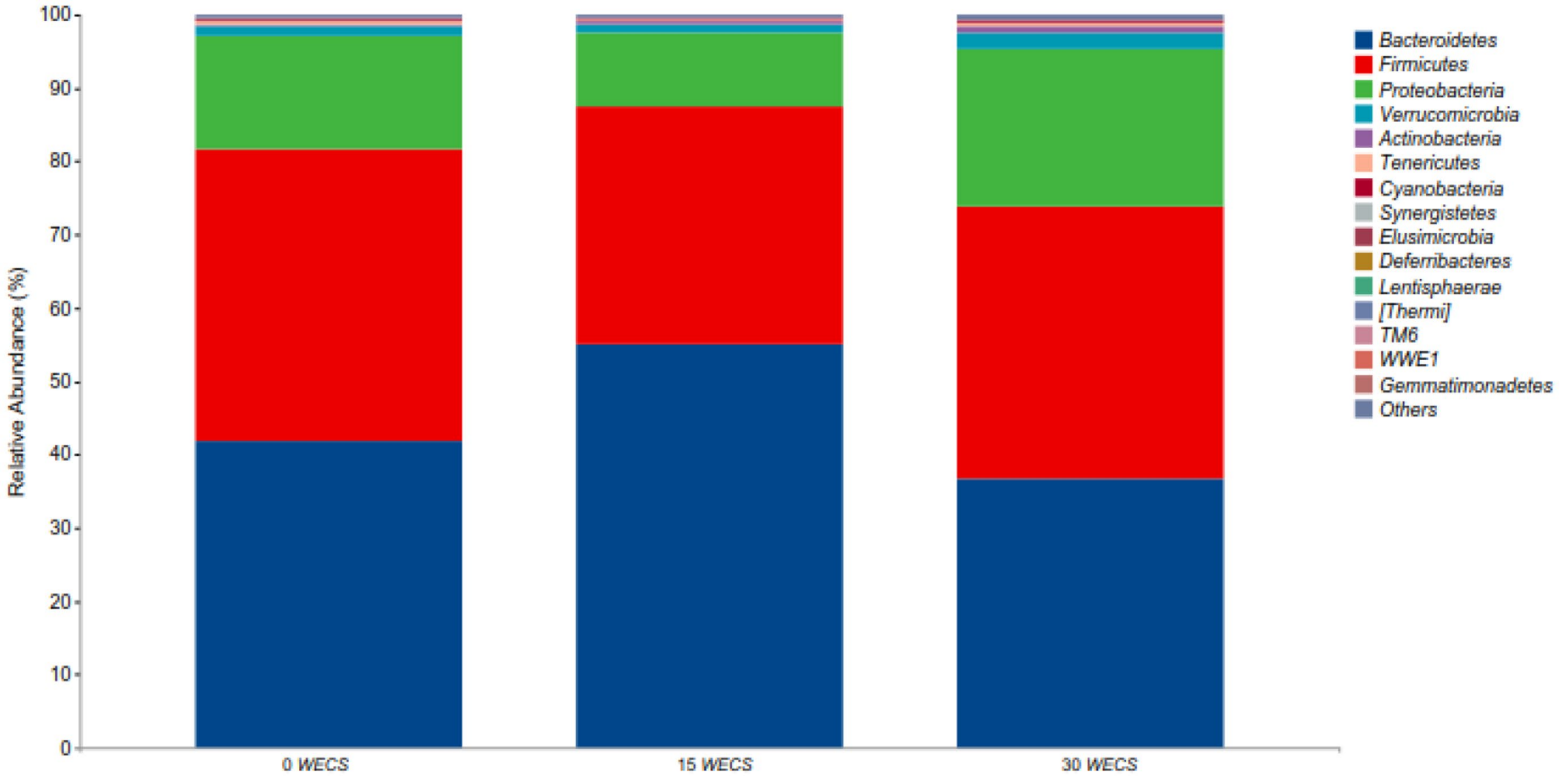
Analysis of the composition of cecal contents at the level of phyla (*n* = 8). 0 *WECS* control group, 15 *WECS* 15% whole-plant ensiled corn stalks +85% concentrate feed, 30 *WECS* 30% whole-plant ensiled corn stalks +70% concentrate feed. In the figure, the abscissa is the name of the grouping scheme, and the ordinate is the relative abundance of each taxonomic at the phylum taxonomic level.

Figure 2 shows the relative abundance at phylum level in each group, and it can be found that the dominant bacteria groups in the cecum of geese in each group are Bacteroidetes, Firmicutes, Proteobacteria, Actinobacteria. Table 8 shows the phyla with differences between groups, with a significant increase in Bacteroidetes abundance in the 15 *WECS* group compared to the 0 *WECS* and 30*WECS* groups (*p* < 0.01).

**Table 8:** Analysis of the composition of cecal contents at the level of genus (*n* = 8).

Items	Diet treatment			value of *p*
	0 *WECS*	15 *WECS*	30 *WECS*	
Bacteroidetes, (%)	41.79 ± 3.21^B^	54.94 ± 2.41^A^	36.78 ± 2.15^B^	*p*<0.001
Firmicutes, (%)	39.88 ± 3.15	35.14 ± 1.10	37.13 ± 3.00	0.477
Proteobacteria, (%)	15.39 ± 2.33	9.96 ± 2.53	21.50 ± 5.08	0.094
Verrucomicrobia, (%)	0.25 ± 0.13	1.25 ± 0.61	0.63 ± 0.36	0.284
Actinobacteria, (%)	0.27 ± 0.07	0.24 ± 0.07	0.27 ± 0.06	0.938
Tenericutes, (%)	0.45 ± 0.15	0.24 ± 0.04	0.41 ± 0.10	0.332
Cyanobacteria, (%)	0.05 ± 0.02	0.13 ± 0.05	0.31 ± 0.12	0.090
Elusimicrobia, (%)	0.06 ± 0.02	0.05 ± 0.02	0.12 ± 0.05	0.229
Others, (%)	0.52 ± 0.07	0.40 ± 0.03	0.40 ± 0.06	0.200

0 *WECS* control group, 15 *WECS* 15% whole-plant ensiled corn stalks +85% concentrate feed, 30 *WECS* 30% whole-plant ensiled corn stalks +70% concentrate feed.

^A–B^ Means with different superscript letters in the same row differ extremely significantly (*p* < 0.01).

### Composition of cecal contents at the level of genus

**Figure 3:**
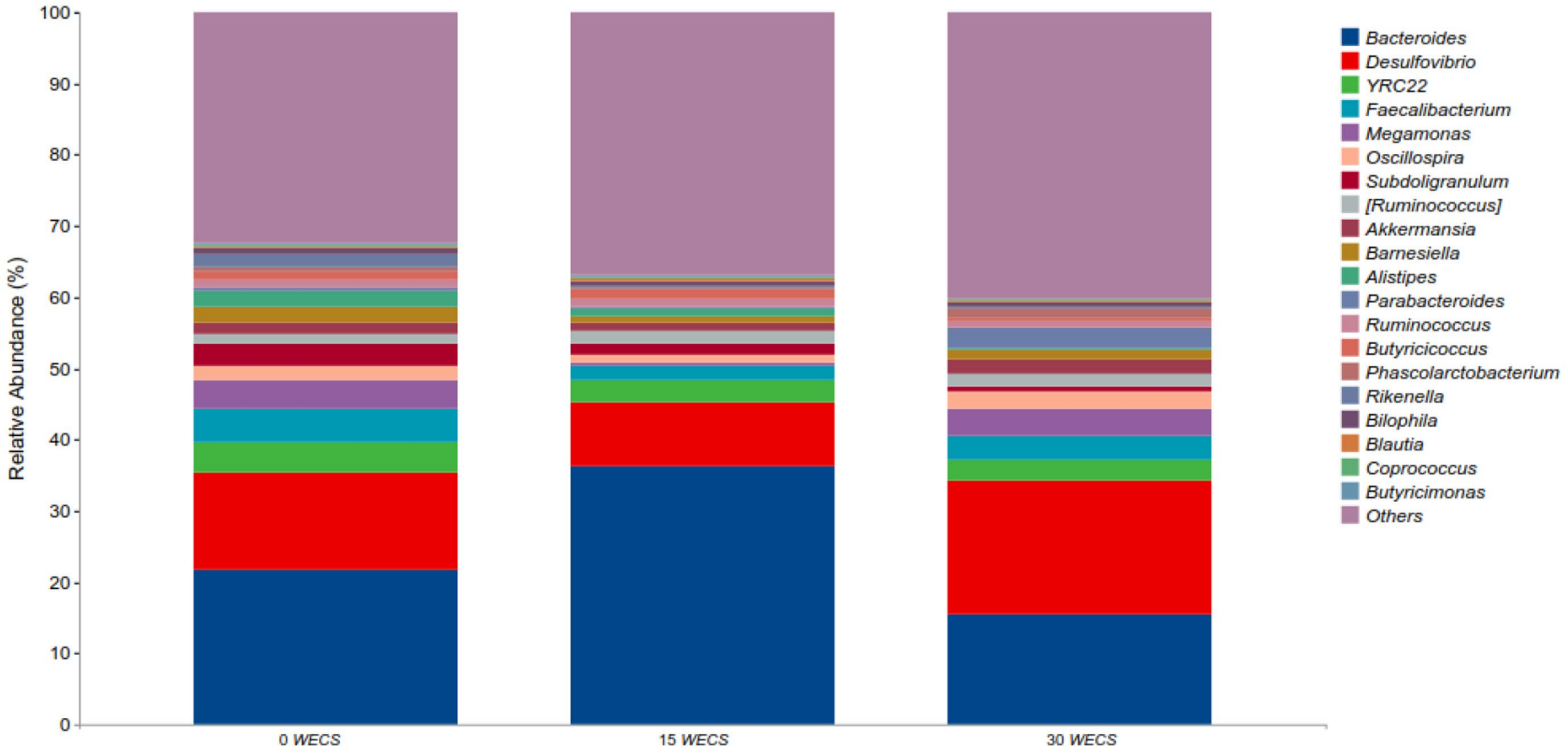
Analysis of the composition of cecal contents at the level of genus (*n* = 8), 0 *WECS* control group, 15 *WECS* 15% whole-plant ensiled corn stalks +85% concentrate feed, 30 *WECS* 30% whole-plant ensiled corn stalks +70% concentrate feed. In the figure, the abscissa is the name of the grouping scheme, and the ordinate is the relative abundance of each taxonomic at the genus taxonomic level.

Figure 3 displays the relative abundance of each group at the genus level, with *Bacteroides*, *Desulfovibrio, YRC22*, and *Faecalibacterium* as the dominant genera. Table 9 highlights some genera with significant differences between groups. Compared to group 0 *WECS*, the abundance of *Rikenella* and *Alistipes* in groups 15 *WECS* and 30 *WECS* was significantly lower (*p* < 0.01). The abundance of *Bacteroides* in group 15 *WECS* was significantly higher than in groups 0*WECS* and 30 *WECS* whereas the abundance of *Butyricicoccus* was significantly higher in group 15 *WECS* (*p* < 0.01). Moreover, the abundance of *Parabacteroides* in the 30 *WECS* group was significantly higher than that in the 0 *WECS* and 15 *WECS* groups (*p* < 0.01).

**Table 9:** Analysis of the composition of cecal contents at the level of genus (*n* = 8).

Item	Diet treatment			value of *p*
	0 *WECS*	15 *WECS*	30 *WECS*	
*Bacteroides*, (%)	21.67 ± 4.11^B^	36.37 ± 2.43^A^	15.48 ± 2.83^B^	*p*<0.001
*Desulfovibrio*, (%)	13.62 ± 2.52	8.94 ± 2.63	18.68 ± 4.71	0.159
*YRC22*, (%)	4.32 ± 1.24	3.01 ± 0.56	3.01 ± 0.76	0.501
*Faecalibacterium*, (%)	4.66 ± 1.12	1.35 ± 0.70	3.28 ± 0.73	0.149
*Megamonas*, (%)	1.58 ± 0.91	0.42 ± 0.38	1.54 ± 0.88	0.431
*Oscillospira*, (%)	2.02 ± 0.35	1.24 ± 0.30	2.60 ± 0.55	0.089
*Subdoligranulum*, (%)	3.22 ± 1.39	1.50 ± 0.59	0.61 ± 0.15	0.665
*[Ruminococcus]*, (%)	0.92 ± 0.20	1.74 ± 0.49	1.49 ± 0.22	0.383
*Akkermansia*, (%)	0.25 ± 0.13	1.25 ± 0.61	0.52 ± 0.37	0.338
*Barnesiella*, (%)	2.55 ± 1.48	0.79 ± 0.26	1.34 ± 0.44	0.497
*Alistipes*, (%)	2.21 ± 0.47^A^	1.18 ± 0.10^B^	0.35 ± 0.05^C^	*p*<0.001
*Parabacteroides*, (%)	0.50 ± 0.12^B^	0.23 ± 0.06^B^	2.88 ± 0.93^A^	0.003
*Ruminococcus*, (%)	1.10 ± 0.29	1.12 ± 0.13	0.92 ± 0.16	0.251
*Butyricicoccus*, (%)	1.13 ± 0.32	0.89 ± 0.17	0.47 ± 0.04	0.149
*Phascolarctobacterium*, (%)	0.77 ± 0.18	0.30 ± 0.07	1.25 ± 0.62	0.237
*Rikenella*, (%)	1.68 ± 0.44^B^	0.17 ± 0.05^A^	0.31 ± 0.13^A^	0.003
*Bilophila*, (%)	0.17 ± 0.05	0.55 ± 0.24	0.61 ± 0.40	0.299
*Blautia*, (%)	0.32 ± 0.07	0.41 ± 0.27	0.15 ± 0.03	0.058
*Coprococcus*, (%)	0.13 ± 0.07	0.29 ± 0.12	0.21 ± 0.03	0.134
*Butyricimonas*, (%)	0.20 ± 0.02^AB^	0.25 ± 0.05^A^	0.12 ± 0.02^B^	0.007
Others, (%)	32.43 ± 1.47	36.90 ± 2.53	36.50 ± 1.47	0.094

0 *WECS* control group, 15 *WECS* 15% whole-plant ensiled corn stalks +85% concentrate feed, 30 *WECS* 30% whole-plant ensiled corn stalks +70% concentrate feed.

^a–b^ Means with different superscript letters in the same row differ significantly (*p* < 0.05).

### Analysis of The distance matrix and PCoA

**Figure 4:**
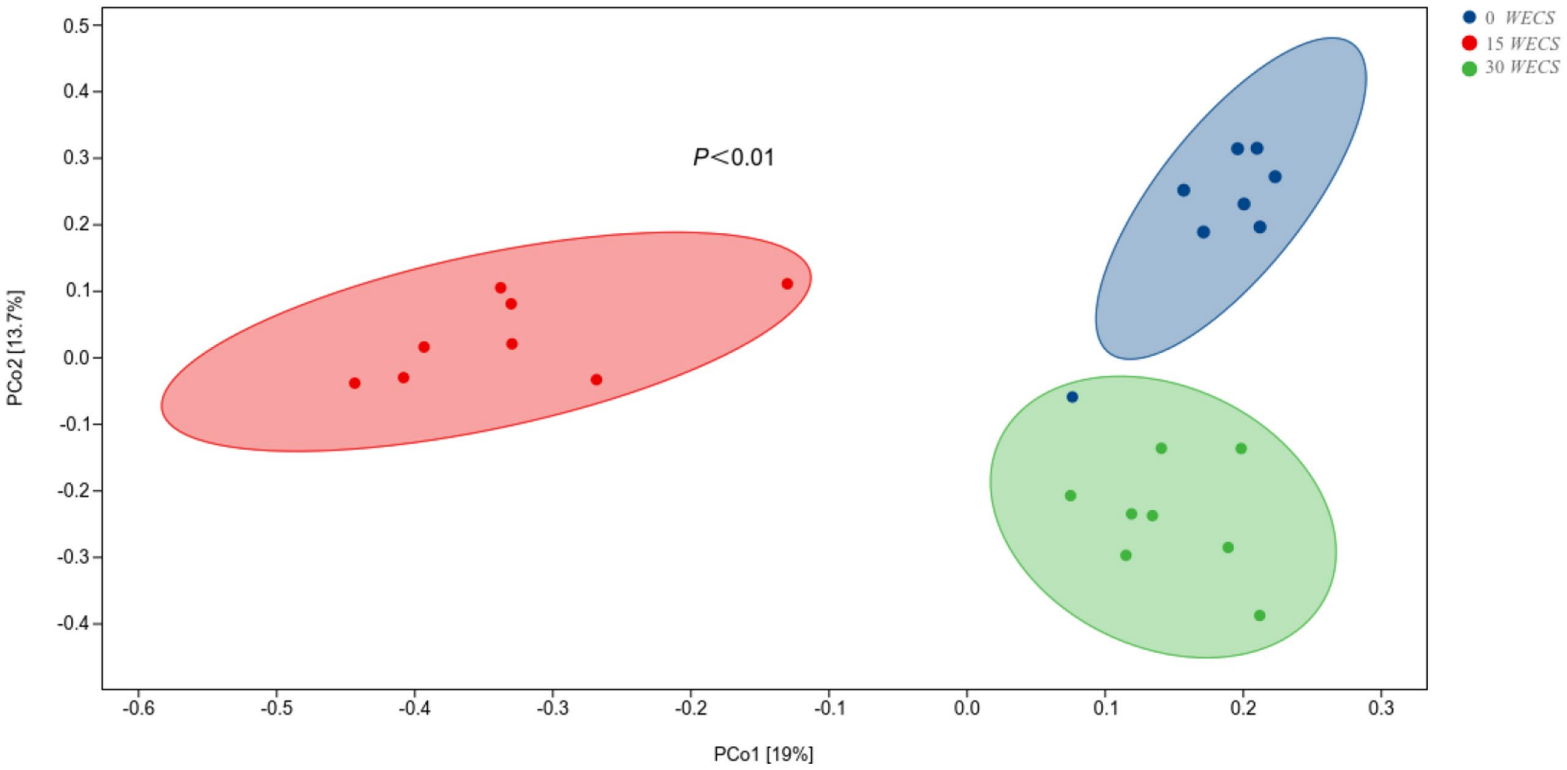
Principal coordinate analysis (PCoA) on community structures of the cecal microbiota after silage corn treatment (*n* = 8), 0 *WECS* control group, 15 *WECS* 15% whole-plant ensiled corn stalks +85% concentrate feed, 30 *WECS* 30% whole-plant ensiled corn stalks +70% concentrate feed. Each point in the figure represents a sample, and different colored points indicate different groupings. The percentages in the brackets on the axes represent the proportion of the sample difference data (the distance matrix) that can be explained by the corresponding axes.

The results of Principal coordinates analysis (PCoA) based on Bray-Curtis algorithm are shown in Figure 4, where Pco1 value is 19% and Pco2 value is 13.7%. The projection of inter-group samples on the matrix is far away, while intra-group samples are relatively clustered together, indicating that the community composition of each group of samples in the corresponding dimension is not similar.

### Correlation analysis

**Figure 5:**
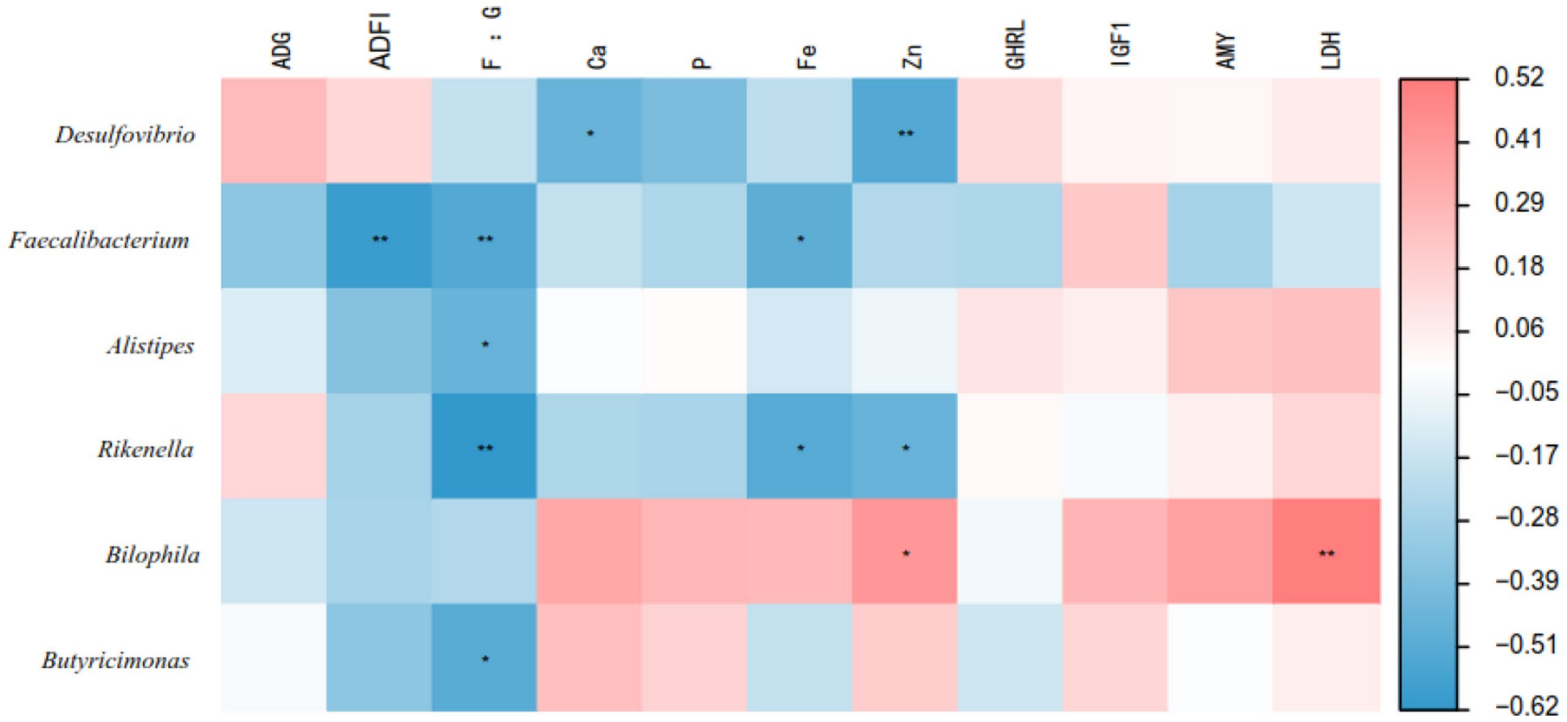
Relationships between growth performance, blood parameters and the top 20 genera in the cecum. Red represents positive correlation, blue represents negative correlation, and *p < 0.05 and **p < 0.01 represent significant correlations.

Figure 5 illustrates the correlation analysis between the bacterial genera of the cecal microbiota of geese and their growth performance and serum parameters. The results showed that *Faecalibacterium* was negatively correlated with ADFI (*p* < 0.05). In addition, *Faecalibacterium*, *Alistipes*, *Rikenella* and *Butyricimonas* were negatively correlated with the F/G ratio (*p* < 0.05). *Faecalibacterium* and *Rikenella* were negatively correlated with serum Fe levels (*p* < 0.05). *Desulfovibrio* and *Rikenella* were negatively correlated with serum Zn levels (*p* < 0.05), while *Bilophila* was positively correlated with serum Zn levels (*p* < 0.05). *Bilophila* was positively correlated with serum LDH levels (*p* < 0.05).

## Discussion

Whole-plant ensiled corn stover (*WECS*) is produced by chopping and ensiling the entire corn plant, including the stover, with the addition of a fermentation inoculant in a sealed container to allow for anaerobic fermentation (16). This process creates a low pH environment that promotes the growth of beneficial bacteria while inhibiting the proliferation of spoilage organisms (17). Geese fed on *WECS* not only directly absorb and utilize existing organic acids, but also promote the colonization of beneficial bacteria in their gastrointestinal tract. Moreover, *WECS* is rich in crude fiber, which can be partially digested and utilized, thereby increasing feed conversion rate and improving intestinal health. During the season of low availability of fresh green forage, *WECS* can provide animals with a stable source of nutrients and microbial biomass, and its high quality as a silage feed has been widely applied in animal production. Zhang et al. (18) showed that feeding *WECS* to fattening bulls increased their average daily weight gain, reduced production costs, and improved rumen degradable protein and fermentation efficiency. Wu et al. (19) found that compared to whole-plant sorghum silage, feeding *WECS* significantly improved lamb growth performance and carcass traits. He et al. (20) found that compared to other crop straws, *WECS* could increase the activity of proteases in the gastrointestinal tract of geese, indicating that *WECS* can improve protein digestion in geese. The results of this experiment showed that feeding *WECS* significantly increased the average daily feed intake of Holdobagy, and adding 15% WECS increased the F/G of geese, indicating that WECS has good palatability for geese, and its acid aroma can enhance their appetite. Although the digestibility and utilization of *WECS* by geese is not high, resulting in an increase in F/G, feeding *WECS* does not affect the FBW and ADG of geese, which is consistent with the results of (21). From an economic point of view, although adding *WECS* may increase feed consumption in the goose farm, it will not have a negative impact on the growth of meat geese, and the price of *WECS* is less than half that of ordinary corn. Therefore, feeding *WECS* can improve the economic benefits of the farm, and the amount of *WECS* added should not be too small.

Fe is a major component of hemoglobin and myoglobin in the body, as well as an important component of various enzymes involved in physiological processes such as oxygen transport, immunity, and metabolism (22). Zn is an important cofactor for various enzymes and is involved in various metabolic and physiological processes such as protein synthesis, DNA synthesis, and maintenance of the immune system (23). Our research results show that feeding *WECS* affects the levels of Fe and Zn in goose serum, with the level of Fe in goose serum positively correlated with the addition level of *WECS*, and the level of Zn in goose serum negatively correlated with the addition level of *WECS*. This may be due to the stimulation of high levels of fiber and lignin in *WECS*, which continuously stimulate the proventriculus to secrete gastric acid, which aids in the digestion of iron, while possibly inhibiting the absorption of Zn, leading to an increase in Fe levels and a decrease in Zn levels in goose serum (24). In addition, the cellulose and anti-nutritional factor phytic acid in *WECS* may form complexes with zinc, reducing its bioavailability. Lactate dehydrogenase (LDH) is an enzyme that catalyzes reactions in animals and is widely present in the cytoplasm. LDH can catalyze the oxidative decomposition of lactate to provide energy for the body (25). The results of this experiment show that after feeding *WECS*, the level of LDH in goose serum significantly decreased and was negatively correlated with the addition level of *WECS*. The reason for the decrease in LDH may be due to the rapid synthesis and consumption of lactate. There are few nutrients in *WECS* feed that can be directly digested and absorbed by geese, and structural polysaccharides such as cellulose and hemicellulose need to be fermented and metabolized by microorganisms to produce organic acids such as lactate. The lactate is then directly provided to intestinal microorganisms and animal cells for oxidative decomposition to produce pyruvate and ATP, and the resulting pyruvate continues to participate in the tricarboxylic acid cycle to provide energy for the body, compensating for the low nutrient concentration in *WECS* feed to maintain normal goose life activities (26). In addition, the decrease in Zn levels in goose serum after feeding *WECS* may also be one of the reasons for the decrease in LDH levels. Zn is an important component of LDH, maintaining its stability and catalytic activity, and the decrease in serum Zn levels may also lead to a decrease in LDH levels (27). The decrease in LDH levels indicates that geese have a considerable digestive ability for *WECS*, but it should be noted that in diets with high proportions of *WECS*, additional Zn should be added to meet the needs of geese.

Principal Coordinates Analysis (PCoA) results showed two-dimensional projected distances indicating differences in bacterial community between samples with different *WECS* ratios. This suggests that varying *WECS* addition ratios could have an impact on the microbial composition of the goose cecum. Alpha diversity analysis indicated that the cecal microorganisms of geese in the 30*WECS* group had higher observed species, Shannon, and Pielou’s evenness indexes, suggesting that increasing the proportion of *WECS* could enhance the richness, uniformity, and diversity of goose cecal microorganisms. These findings are consistent with the results of Chao et al. (28).

Similar to previous studies, this experiment found that the phyla Bacteroidetes, Firmicutes, and Proteobacteria were the dominant bacterial phyla in the cecum of geese. Although adding *WECS* did not change the dominant bacterial phyla, it did affect their relative abundance (29, 30). Bacteroidetes is a gram-positive bacterial phylum associated with weight gain and feed intake (31, 32). The results of this experiment showed that the abundance of Bacteroidetes increased and then decreased with the increasing proportion of *WECS* addition. This may be due to the nutritional concentration of the feed and the digestive characteristics of the goose. Adding *WECS* can reduce the nutritional concentration of the feed, forcing geese to increase their feed intake to maintain their energy levels. At low addition proportions, the feed intake of geese increases, causing the food renewal rate in their intestines to accelerate. However, the cecum is not sufficiently adapted to the cellulose in *WECS*, which reduces the efficiency of digestion and absorption of feed nutrients and slows down weight gain. An increase in feed intake can increase the abundance of Bacteroidetes (33). However, as the proportion of *WECS* increases, more organic acids enter the goose’s intestines, which can be directly digested and utilized. The renewal rate of food in the intestine slows down, and the cecum becomes more adapted to the cellulose in *WECS* (34). This leads to an increase in the digestion and absorption rate of cellulose and feed, a decrease in feed intake, and a decrease in the abundance of Bacteroidetes (35). Additionally, the study found a positive correlation between the relative abundance of Cyanobacteria and the proportion of *WECS* addition, but the difference was not significant. The increase in abundance of Cyanobacteria may be due to the residual cyanobacteria on *WECS* entering the cecum of the digestive tract (7).

*Alistipes* is a genus of bacteria belonging to the family Bacteroidetes, which is widely found in the cecum of geese (10, 36). Our experimental results indicate that the abundance of *Alistipes* is negatively correlated with the proportion of *WECS* in the feed, suggesting that this bacterium avoids a diet rich in plant-based food and grows well in a low-fiber diet (37). Kong et al. (38) found that the abundance of *Alistipes* in the cecum of mice fed a high-fat, high-sugar diet increased significantly, which is consistent with our results. Studies suggest that *Alistipes* may play an important role in the breakdown of complex carbohydrates and the production of SCFAs, which can be used as a source of energy for the host. The abundance of *Alistipes* may be related to the concentration of starch in the cecum (39). Unlike geese fed concentrated feed, the starch concentration in the cecum contents of geese fed *WECS* is lower, resulting in a decrease in the abundance of *Alistipes*. *Rikenella* is a gram-negative rod-shaped bacterium and a member of the *Rikenellaceae* family, found in the cecum and feces of birds. Our research results indicate that the level of *Rikenella* in goose cecum was significantly reduced after feeding with *WECS*, consistent with the findings of (18). The decrease in *Rikenella* abundance may be attributed to its narrow metabolic capability. The main carbon source for *Rikenella* growth is glucose, and it can hardly hydrolyze the fiber polysaccharides in *WECS*, which may be the reason for its reduced abundance in the cecum (40). *Parabacteroides* is a genus of bacteria belonging to the family *Bacteroidaceae*, which is a common component of the human and animal gut microbiota. Currently, there is limited research on the role of *Parabacteroides* in the goose gut, but it has been shown to be one of the main species involved in the digestion of dietary fiber in the human gut, producing SCFAs that maintain gut health (41, 42). Our experimental results show that adding 30% *WECS* to the feed significantly increases the abundance of *Parabacteroides* in the goose cecum. Therefore, the abundance of *Parabacteroides* is positively correlated with the dietary fiber level in the feed. *Butyricimonas* is a Gram-negative anaerobic bacterium belonging to the family *Lachnospiraceae*, which ferments dietary fiber to produce butyric acid, providing energy to intestinal wall cells and maintaining a healthy gut microbiota, preventing enteritis (43). Our experimental results indicate that adding 15% ensiled corn significantly increases the abundance of *Butyricimonas* in the goose cecum. However, when the addition amount reaches 30%, the abundance of *Butyricimonas* decreases. This may be related to the pH of the ensiled corn feed, as high addition amounts can lower the pH of the feed and have an adverse effect on the cell wall of *Butyricimonas* (44).

## Conclusion

In summary, adding *WECS* to the diet may increase feed consumption in geese, but it does not have a negative impact on their growth and can increase economic benefits. However, it is important to pay attention to the amount of *WECS* added and supplement the diet with Zn to meet the geese’s normal needs. The proportion of *WECS* in the diet does not appear to affect the major bacterial phyla in the goose cecum, but it does impact the overall microbial composition and abundance of certain bacterial phyla and genera. Increasing the proportion of *WECS* in the feed can enhance the richness, evenness, and diversity of the cecal microbiota. As the amount of *WECS* added to the diet increases, the abundance of *Alistipes* decreases. On the other hand, the abundance of *Parabacteroides* increases with an increasing proportion of WECS, while the abundance of *Butyricimonas* increases when 15% *WECS* is added and decreases when 30% *WECS* is added to ensiled corn.

## Methods

All animal protocols were approved by the Shanghai Science and Technology Committee (STCSM) with a license number of SYXK (HU): 2015–0007, and carried out in accordance with approved guidelines and regulations. Every effort was made to minimise the suffering of the geese in the current study.

## Data availability statement

The datasets presented in this study can be found in online repositories. The names of the repository/repositories and accession number(s) can be found below: https://www.ncbi.nlm.nih.gov/bioproject; PRJNA961934.

## Ethics statement

The animal study was reviewed and approved by the Animal Care and Use Committee of the Shanghai Academy of Agricultural Sciences (SAASPZ0522046), Shanghai Academy of Agricultural Sciences.

## Author contributions

XW completed the experimental manuscript’s initial draft writing and inspection. HW and DH carried out the experimental design and paper editing. YY, GL, CW, and YL performed the data software analysis and sample collection. SG participated the on-site breeding of geese. All authors mentioned above have reviewed and approved the final manuscript.

## Funding

This study was financially supported by the Climbing plan of Shanghai Academy of Agricultural Sciences [PG23171], the Earmarked Fund for China Agriculture Research System [CARS-42-35] and the SAAS Program for Excellent Research Team (SPERT), and the Special Project of Modern Agriculture [ZY221702].

## Conflict of interest

The authors declare that the research was conducted in the absence of any commercial or financial relationships that could be construed as a potential conflict of interest.
